# Human and Bovine Tuberculosis

**Published:** 1903-05-02

**Authors:** 


### The Hospital.

A Journal of

The Medical Sciences and Hospital Administration.

Edited by Sir Henry Burdett, K.C.B.

Vol. XXXIV.—No. 866. [May 2, 1903.

### Human and Bovine Tuberculosis.

Our readers will remember the feeling of surprise, not altogether unmingled with incredulity, which was excited by the announcement, during the British Congress on Tuberculosis in 1901, of Professor Koch's disbelief in the identity of human and bovine tuberculosis, and of his opinion that the precautions which had been based upon the supposition of identity were superfluous and might be abandoned. As a result of experiments conducted upon nineteen animals he "felt justified" in maintaining that human tuberculosis differed from bovine, and could not be transmitted to cattle. The inference was, of course, that transmission in the opposite direction was also impossible, and that tuberculous flesh and tuberculous milk were no longer to be regarded as dangerous to human consumers. The announcement was of so surprising a character that the Professor, in making it, laid stress upon the manifest necessity for a repetition of his experiments by other observers.

The gauntlet thus thrown down to pathologists was at once accepted by inquirers in different countries, and of these the first to bring forward definitely attained results have been Professor David Hamilton and Mr. M'Lauchlan Young, working together in the Agricultural Department of the University of Aberdeen. An account of their experiments was laid before the Highland and Agricultural Society of Scotland, and has just been published as a reprint from the Transactions. It contains detailed accounts of experiments upon 20 animals, arranged in four series for the elucidation of successive points, and it must, we think, be accepted as absolutely conclusive of the communicability of human tuberculosis to bovine animals. In nearly every case the experimenters succeeded where Professor Koch had failed.

The first series of experiments, in which six young calves were fed with milk to which human tuberculous sputum had been added, were inconclusive in the case of two, which died prematurely; but in the remaining four tuberculous infection was found either in the tonsils or in the lymph glands in connection with some part of the alimentary tract. The mesenteric glands, in particular, were much involved; they were enlarged, and showed a tubercular giant-cell structure which reproduced the disease on re-inoculation into guinea-pigs. Each of the animals had been fed on 18 occasions with tubercular sputum from a single patient, and they were slaughtered for examination after from 65 to 69 days. Their condition showed that the tubercle bacillus is able to pass from the alimentary canal to the adjacent glands without the production of any discoverable lesion of the mucous membrane, and therefore strongly supports the belief that the ordinary mesenteric tubercle of children is really due, as has been supposed, to infection conveyed through the ingesta. In the second series of experiments the object was to ascertain whether the human disease could be communicated to the calf by subcutaneous inoculation. In one of the first six the result was negative or uncertain; but the remaining five calves all became infected, and their tuberculosis was reproduced by inoculation into guinea-pigs. The third series of experiments included the repetition of some already made. The fourth series included two experiments designed to show whether or not the human bacillus gains in virulence by being transferred from one bovine host to another. Calves a few days old were inoculated subcutaneously with fragments of tubercular lymph-glands removed from the carcasses of two other calves which had previously been inoculated successfully from human cervical glands. Each of these animals developed a widespread tuberculosis far more virulent than that of the primary host, clearly demonstrating that the human bacillus, once ingrafted on the calf, becomes acclimatised to its new surroundings, and has its power of inducing lesions enhanced. At the close of their paper, the authors formulate ten conclusions which their experiments appear to justify. The first opens with what may be described as a golden bridge for Professor Koch, in the shape of a declaration that "although human tubercle is probably not so virulent for the calf as that derived from bovines," yet it can be readily inoculated upon that animal. The ninth is that the facts go to favour the view that the human bacillus and that of bovines are identical, but modified somewhat by their environment; and the tenth is that the results obtained "are a direct contradiction of those alleged to have been obtained by Koch and Schütz." It is hardly necessary to add that the investigation appears to have been worked out, in every detail, with the strictest care and with the most absolute completeness. The calves employed were young, were carefully tested with tuberculin before being inoculated, and were kept with the strictest precautions. The shed in which they were housed was new and freshly lime-washed; the milk on which they were fed was furnished by cows which had been tested with tuberculin and failed to react; and in every conceivable way external infection was excluded. We think there can be no doubt that Professor Koch's assumption of a difference between the human and the bovine bacilli must be finally and completely abandoned.

